# Differential Impacts of pH and Acid Type on Seed Germination and Seedling Growth in *Brassica juncea* and *Raphanus sativus*

**DOI:** 10.1002/pei3.70074

**Published:** 2025-09-06

**Authors:** Mythri Vallabhaneni, Zhongmin Dong, Ellie M. Goud

**Affiliations:** ^1^ Department of Biology Saint Mary's University Halifax Nova Scotia Canada

**Keywords:** acetic acid, acid rain, hydrochloric acid, plant growth, plant stress, seed germination, sulfuric acid

## Abstract

Elevated acidity from natural and anthropogenic sources can be a significant stressor for plants, affecting essential processes such as nutrient uptake and growth. While low pH (< 4) is generally considered stressful for plants, differential impacts of distinct acid types—organic versus inorganic, strong versus weak—on plant growth and development remain unclear. To address this knowledge gap, we evaluated the responses of two Brassicaceae species to organic (acetic) and inorganic (hydrochloric, sulfuric) acids at three pH levels (pH 2.5–5.5) to determine whether acid responses are solely pH‐dependent or if acid type specificity plays a role in plant functional responses, thereby providing insights into the mechanisms of acid responses and tolerance. Plant responses varied based on acid types, even at the same pH levels. Hydrochloric acid promoted higher seed germination but hindered seedling growth and morphological development of roots, shoots, and leaves, while acetic and sulfuric acids had the opposite effects. These results highlight the influence of acid types on plant functions, specifically affecting distinct developmental stages. These findings suggest that plant responses to acid stress are not solely pH‐dependent but are significantly influenced by the specific chemical properties of the acids involved, providing valuable insights for managing acid‐impacted wild plant communities.

## 1 Introduction

Acidic conditions, whether arising from natural or anthropogenic sources, pose a significant stressor to plants, impacting essential physiological processes such as nutrient uptake and growth (Shavrukov and Hirai 2016; Zhang et al. 2015; Zhu and Shen 2023). While acid rain, largely resulting from industrial emissions, represents a major source of anthropogenic acid (Singh and Agrawal 2008), naturally occurring acidity is also prevalent in poorly buffered soils and environments high in organic acids from biological activity, such as peat bogs (Bowman et al. 2014; Goud and Roddy 2022). These habitats challenge plants to develop stress tolerance strategies or avoidance mechanisms (Bandurska 2022).

Traditional research has primarily focused on soil pH as the key determinant of plant stress in acidic environments (Borhannuddin Bhuyan et al. 2019; Lu et al. 2020; Zhang et al. 2023). However, the specific effects of different acid types—whether organic or inorganic, strong or weak—on plant growth and function remain underexplored. Distinguishing between the effects of organic versus inorganic acids and strong versus weak acids is crucial, particularly in environments where different acids may co‐occur or fluctuate in concentration. Acid types such as sulfuric acid (H_2_SO_4_), hydrochloric acid (HCl), and acetic acid (CH_3_COOH) are common in both natural and human‐altered environments, yet their effects on seed germination and plant growth have not been systematically compared under controlled conditions.

We sought to fill this gap by evaluating plant responses to different acids at various pH levels, providing insights into the broader implications of acid stress for wild plant communities. We selected sulfuric acid (H_2_SO_4_), hydrochloric acid (HCl), and acetic acid (CH_3_COOH) to represent a range of strong and weak acids present in acid rain or naturally acidic environments. Sulfuric acid (H_2_SO_4_) and hydrochloric acid (HCl) are strong inorganic acids commonly associated with anthropogenic activities such as industrial emissions and the formation of acid rain (Evans et al. 2011; Grennfelt et al. 2020). When dissolved in water, H_2_SO_4_ fully dissociates into hydrogen (H^+^) and sulfate (SO_4_^2−^) ions, while HCl fully dissociates into H^+^ and chloride (Cl^−^) ions (Heald 1896; Speight 2017). Acetic acid (CH_3_COOH), a weak organic acid, is commonly found in naturally acidic environments where organic matter decomposes and releases low molecular weight organic acids (Adeleke et al. 2017). In solution, CH_3_COOH only partially dissociates into H^+^ and acetate ions (CH_3_COO^−^), contributing to a less drastic pH reduction compared to strong acids like H_2_SO_4_ or HCl (Chatveera and Lertwattanaruk 2014).

We measured seed germination rates, seedling growth, and seedling morphological traits (leaf area, shoot length, and root length) to determine the mechanisms by which different acids influence plant responses. We used two fast‐growing species from the Brassicaceae family, *Brassica juncea* (brown mustard) and *Raphanus sativus* (daikon radish), as model organisms due to their rapid growth in laboratory settings and naturally different acid tolerances (Serdyuk et al. 2022; Singh 2021). *Brassica juncea* is a cool weather plant that thrives predominantly in temperate climates and is mildly acid‐tolerant (Serdyuk et al. 2022; Shekhawat et al. 2012) while *Raphanus sativus* grows in various climate zones from temperate to tropical regions and is not considered acid tolerant (Singh 2021).

## 2 Materials and Methods

We grew plants hydroponically under a factorial design with three main factors: acid type, pH level, and species. Solutions of each acid were prepared at three pH levels (2.5, 4, 5.5) by diluting the acids with distilled water until the desired pH level was attained. pH values represented a range of acidic conditions from extreme acidity (pH 2.5–4) to conditions found in acid rain and natural ecosystems (pH 5.5). A control using distilled water at pH 7 was included to represent neutral conditions. pH was determined with an Orion Star A211 benchtop pH meter (Thermo Fisher Scientific, Waltham, MA, USA). The total number of treatment solutions, including distilled water as control, was 10 (three pH levels × three acid types plus one water control).

To assess seed germination, 30 seeds of each species were placed in individual Petri dishes lined with moist paper and treated with 1 mL of acid solution or distilled water (*n* = 3 dishes, per species, per treatment). The dishes were sealed with parafilm to maintain moisture, and seeds were monitored daily for 10 days. An additional 0.3 mL of solution was added every 3–4 days as required to maintain moisture. Germination rates were recorded after 10 days since starting acid treatments as the percentage of seeds that successfully germinated relative to the total number of seeds. The seeds used in this experiment were organic sprouting seeds from Mumm's Sprouting Seeds (Parkside, Saskatchewan, Canada).

To assess seedling growth and morphological development, germinated seeds of similar hypocotyl length were transferred to CYG seed germination pouches (Mega International, Roseville, MN, USA) and filled with 20 mL of the respective treatment (*n* = 5 seedlings, per species, per treatment). Seedlings were grown under a 16‐h daylight period at 22°C for 11 days, with an additional 2.5 mL of solution added midway through the experiment to maintain hydration. At the end of the experiment, after 11 days since seedlings were transferred to hydroponics, we measured leaf area, shoot length, root length, and total biomass for each plant. Total leaf area (cm^2^) was measured using a LI‐3000C leaf area meter with a conveyor attachment (LI‐COR, Lincoln, NE, USA). Shoot and root lengths (cm) were measured using a ruler. After morphological measurements, plants were oven‐dried at 70°C for 48 h and weighed for total plant biomass using an analytical balance.

All statistical analyses were performed in R version 4.3.1 (R Core Team 2023). Plant variables (germination rate, biomass, leaf area, shoot, and root length) were assessed for normality using Shapiro–Wilk tests and for homogeneity of variance using Levene's tests. Variables satisfied the analysis of variance (ANOVA) assumptions after square‐root transformation. We assessed the impacts of acid type, pH level, species, and their interaction on plant variables using multi‐factor ANOVA and Tukey post hoc tests.

## 3 Results

Seed germination rate, seedling biomass, leaf area, shoot length, and root length all varied by acid type, pH level, and species. All five variables had an acid type × pH level interaction. Species had a significant interaction with acid type and pH for all variables except germination rate. Seedling biomass, leaf area, and root length also had an acid type × pH level × species interaction (Table 1).

**TABLE 1:** Results from a multi‐factor analysis of variance (ANOVA) with acid type, pH level, species, and their interaction as dependent variables and seed germination rate, seedling biomass (g), leaf area (cm^2^), shoot and root length (cm) as independent variables for daikon radish ( *R. sativus* ) and brown mustard ( *B. juncea* ) (*n* = 3 dishes per species, per treatment) and seedlings (*n* = 5 plants per species, per treatment).

	Df	SS	MeanSS	*F*	*p*
Germination rate					
Acid	3	1.24	0.41	858.48	< 0.0001
pH	2	1.66	0.83	1717.54	< 0.0001
Species	1	0.00	0.003	7.08	0.011
Acid: pH	4	2.24	0.56	1158.17	< 0.0001
Acid: species	3	0.001	0.0004	0.90	0.450
pH: species	2	0.001	0.001	1.48	0.239
Acid: pH: species	4	0.001	0.0003	0.61	0.655
Residuals	40	0.02	0.001		
Biomass					
Acid	3	0.028	0.009	50.23	< 0.0001
pH	2	0.058	0.029	158.19	< 0.0001
Species	1	0.133	0.133	728.61	< 0.0001
Acid: pH	4	0.036	0.009	48.59	< 0.0001
Acid: species	3	0.004	0.001	6.68	0.0004
pH: species	2	0.005	0.003	14.95	< 0.0001
Acid: pH: species	4	0.008	0.002	11.00	< 0.0001
Residuals	80	0.015	0.0002		
Leaf area					
Acid	3	3.37	1.12	52.45	< 0.0001
pH	2	5.89	2.95	137.73	< 0.0001
Species	1	4.38	4.38	204.92	< 0.0001
Acid: pH	4	3.92	0.98	45.87	< 0.0001
Acid: species	3	0.35	0.12	5.46	0.002
pH: species	2	0.24	0.12	5.51	0.006
Acid: pH: species	4	0.30	0.07	3.48	0.011
Residuals	80	1.71	0.02		
Shoot length					
Acid	3	4.06	1.35	36.32	< 0.0001
pH	2	7.56	3.78	101.33	< 0.0001
Species	1	3.45	3.45	92.64	< 0.0001
Acid: pH	4	8.53	2.13	57.18	< 0.0001
Acid: species	3	0.52	0.18	4.69	0.005
pH: species	2	1.49	0.74	19.96	< 0.0001
Acid: pH: species	4	1.58	0.40	10.60	< 0.0001
Residuals	80	2.98	0.04		
Root length					
Acid	3	29.02	9.67	41.48	< 0.0001
pH	2	51.77	25.89	111.01	< 0.0001
Species	1	12.87	12.87	55.20	< 0.0001
Acid: pH	4	36.56	9.14	39.19	< 0.0001
Acid: species	3	9.14	3.05	13.07	< 0.0001
pH: species	2	3.44	1.72	7.38	0.001
Acid: pH: species	4	1.06	0.27	1.14	0.346
Residuals	80	18.66	0.23		

*Note:* All independent variables were square‐root transformed.

**FIGURE 1:**
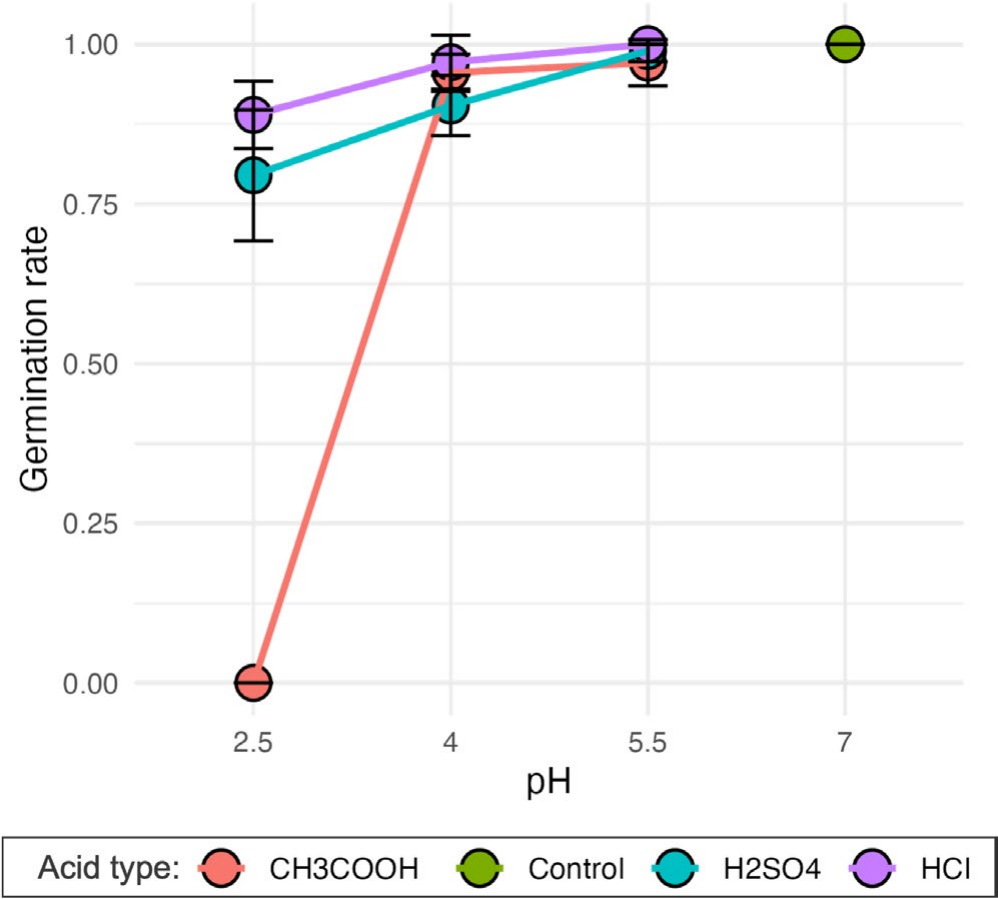
Variation in seed germination rate for *B. juncea* (brown mustard) and *R. sativus* (daikon radish) combined across 10 treatments: Three pH levels for acetic (CH_3_COOH, in red), sulfuric (H_2_SO_4_, in blue) and hydrochloric (HCl, in purple) acids plus a water control (in green). Data are from individual petri dishes containing 30 seeds each (*n* = 3 dishes per species, per treatment) with standard error. Statistical differences among treatments are reported in Tables 1 and 2.

Seed germination rate for both species was 100% in the control and lowest at pH 2.5. The highest overall germination was in hydrochloric acid, which had nearly 100% germination across all pH levels. For acetic acid, germination rates were similar to the control across pH levels 5.5 and 4 (0.94–0.98) and were 0% in pH 2.5. For sulfuric acid, germination rates were also similar to the control for pH 5.5 (0.99), slightly lower in pH 4 (0.87–0.94), and lowest in 2.5 (0.76–0.83; Figure 1; Table 2).

**TABLE 2:** Variation in seed germination rate, seedling biomass (g), leaf area (cm^2^), shoot and root length (cm) across 10 treatments: Three pH levels for acetic (CH_3_COOH), sulfuric (H_2_SO_4_) and hydrochloric (HCl) acids plus a water control. Data are mean values for daikon radish ( *R. sativus* ) and brown mustard ( *B. juncea* ) seeds (*n* = 3 dishes per species, per treatment) and seedlings (*n* = 5 plants per species, per treatment).

Species	Acid type	pH level	Germination	Biomass	Leaf area	Shoot length	Root length
Radish	Water	7	1.00a	0.030a	2.06ab	2.44bc	13.04abc
	CH_3_COOH	5.5	0.98ab	0.025ab	2.06ab	2.86abc	15.07ab
	CH_3_COOH	4	0.97ab	0.021abc	1.33bc	4.48a	12.61abc
	CH_3_COOH	2.5	0.00e	0.00j	0.00g	0.00f	0.00g
	H_2_SO_4_	5.5	0.99a	0.025ab	2.66a	2.51bc	16.93a
	H_2_SO_4_	4	0.94abc	0.019bc	1.05cd	3.13ab	15.76a
	H_2_SO_4_	2.5	0.83 cd	0.014c	0.94cd	1.91bcd	6.70cdef
	HCl	5.5	1.00a	0.021abc	1.06cd	2.55bc	13.58abc
	HCl	4	0.99a	0.014c	0.84cd	2.49bc	11.49abc
	HCl	2.5	0.92abc	0.013cd	0.77cd	2.49bc	10.53abcd
Mustard	Water	7	1.00a	0.006efg	0.91cd	1.58cde	14.11ab
	CH_3_COOH	5.5	0.96ab	0.007ef	1.05cd	1.89bcd	14.15ab
	CH_3_COOH	4	0.94abc	0.002ghi	0.61de	0.66e	4.53def
	CH_3_COOH	2.5	0.00e	0.00j	0.00g	0.00f	0.00 g
	H_2_SO_4_	5.5	0.99a	0.007de	0.89cd	1.83bcd	13.65abc
	H_2_SO_4_	4	0.87abcd	0.005efgh	0.55de	2.21bc	10.42abcd
	H_2_SO_4_	2.5	0.76d	0.002hi	0.48de	1.86bcd	7.67bcde
	HCl	5.5	1.00a	0.003fghi	0.25ef	2.55bc	4.83def
	HCl	4	0.95abc	0.001i	0.12f	0.87de	2.30f
	HCl	2.5	0.86bcd	0.002i	0.08 fg	0.92de	3.27ef

*Note:* Letters correspond to Tukey post hoc groups based on a multi‐factor ANOVA performed using square‐root transformed variables.

**FIGURE 2:**
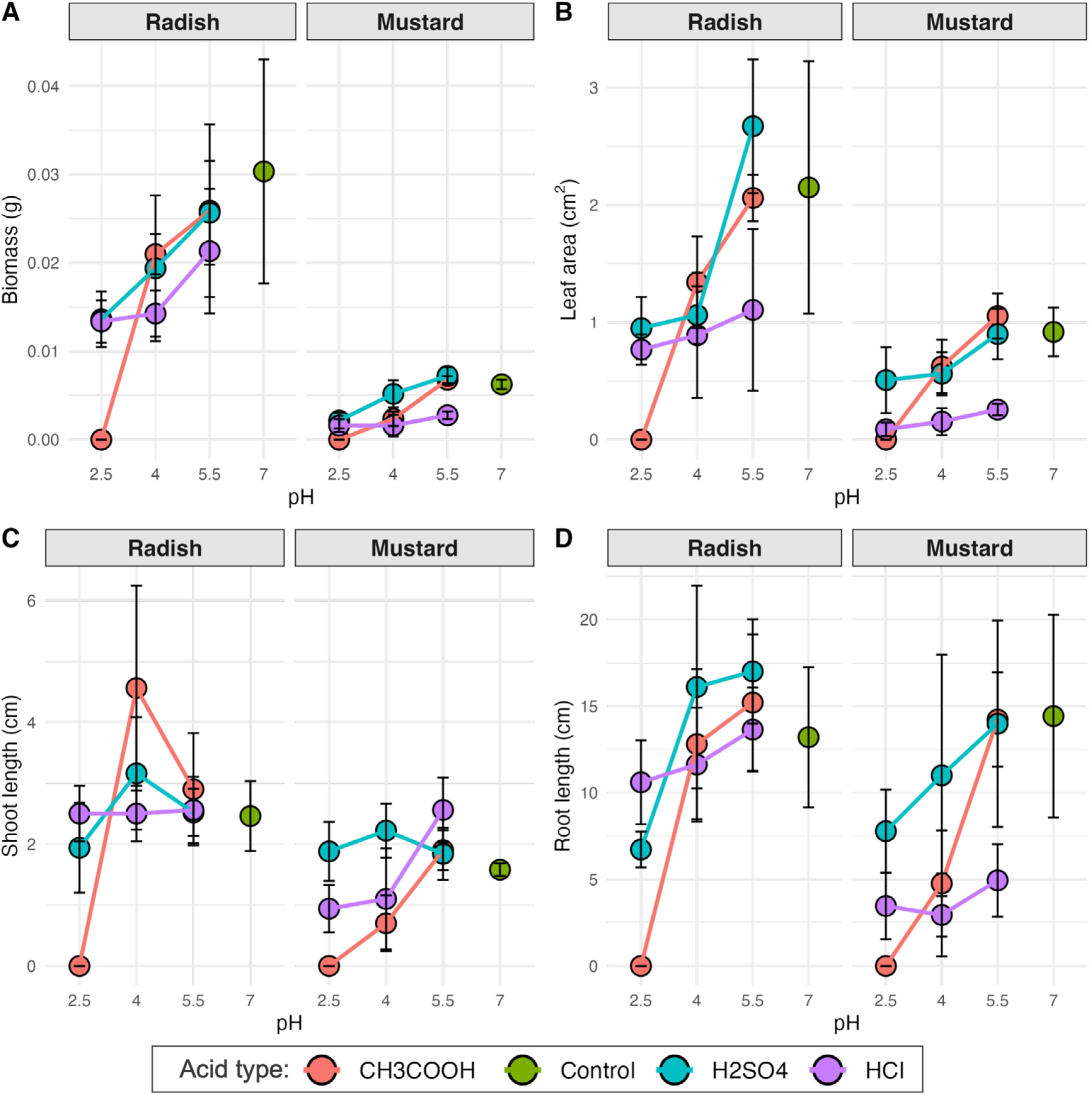
Variation in (A) total biomass, (B) leaf area, (C) shoot length, and (D) root length for brown mustard ( *B. juncea* ) and daikon radish ( *R. sativus* ) across 10 treatments: Three pH levels for acetic (CH_3_COOH, in red), sulfuric (H_2_SO_4_, in blue) and hydrochloric (HCl, in purple) acids plus a water control (in green). Data are from individual plants (*n* = 5 plants per species, per treatment) with standard error. Statistical differences among treatments are reported in Tables 1 and 2.

Seedling biomass increased with pH, with maximal biomass in the water control for radish and in the acetic and sulfuric acids at pH 5.5 for mustard. Biomass was lowest at pH 2.5 across all acids, and there was complete mortality for both species in acetic acid at pH 2.5. For radish, biomass was similar across all three acids at pH 5.5 (0.021–0.025 g). At pH 4, biomass was similar to pH 5.5 for acetic and sulfuric acids but considerably lower in hydrochloric acid (0.014 g). For mustard, biomass was similar for acetic and sulfuric acids at pH 5.5 (0.007 g) and lower for hydrochloric acid (0.003 g). At pH 4, biomass was lowest in acetic acid (0.002 g) and was similarly higher for hydrochloric and sulfuric acids (0.005 g). Biomass was similarly low at pH 2.5 for hydrochloric and sulfuric acids and pH 4 for acetic acid (0.002 g; Figure 2A; Table 2).

Leaf area increased with pH and was consistently smaller in hydrochloric acid relative to acetic and sulfuric acids at all pH levels (Figure 2B; Table 2). Radish displayed the largest leaf area under sulfuric acid at pH 5.5, followed closely by the control and acetic acid at pH 5.5. Mustard showed a similar trend, with the largest leaf area recorded under the control and sulfuric acid at pH 5.5. There were significant reductions in leaf area under acetic and hydrochloric acid at lower pH levels (2.5 and 4).

Variation in shoot length was species‐and acid‐specific; shoot length did not vary with pH for radish grown under hydrochloric acid and was not significantly different from the control. Radish seedlings were tallest at pH 4 under acetic, followed by sulfuric acid, and shortest at pH 2.5 in sulfuric acid. In contrast, mustard shoot length did vary with pH for hydrochloric acid but not for sulfuric acid. Mustard seedlings grown in sulfuric acid were taller than the control across all pH levels. Mustard seedlings were at pH 5.5 in hydrochloric acid and pH 4 in sulfuric acid, followed by pH 5.5 for both acetic and sulfuric acids (Figure 2C; Table 2).

Variation in root length was also species‐and acid‐specific; for radish, the longest roots were observed under sulfuric acid at pH 4 and 5.5, while the shortest were in sulfuric acid at pH 2.5. Root length was similar between acetic and hydrochloric acids at pH 4 and 5.5. Mustard showed longer roots in the control treatment and at pH 5.5 in acetic and sulfuric acids. Root length was lowest under hydrochloric acid and did not vary with pH (Figure 2D; Table 2).

## 4 Discussion

We demonstrate that the effects of acid stress on seedling growth and development are not solely dependent on pH but are also significantly influenced by the chemical nature of the acids themselves. The differential impacts of sulfuric acid (H_2_SO_4_), hydrochloric acid (HCl), and acetic acid (CH_3_COOH) on germination rates, seedling biomass, and morphological traits provide insight into how plants respond to varying types of acid stress.

Sulfuric acid, a strong inorganic acid commonly associated with acid rain and industrial pollution (Zhang et al. 2023; Singh and Agrawal 2008), generally supported higher germination rates and seedling development, particularly at moderate pH levels (4 and 5.5). *Raphanus sativus* (radish) and *B. juncea* (mustard) both exhibited relatively high germination rates under sulfuric acid, with levels similar to the control at pH 5.5 (Figure 1). The sulfate ions (SO_4_^2−^) released upon dissociation of H_2_SO_4_ may provide a nutrient source that mitigates some of the negative impacts of acidity (Narayan et al. 2023). Biomass was maximized under sulfuric acid at pH 5.5 for both species, suggesting that sulfuric acid, at least at this concentration, creates a conducive environment for seedling growth. Additionally, the largest leaf area and longest root lengths were also observed under this treatment (Figure 2), indicating that sulfuric acid may enhance nutrient uptake efficiency without causing significant toxicity. Plants are likely utilizing the low levels of sulfur present in H_2_SO_4_ as essential nutrients, thus benefiting from it and overcoming the negative impact of relatively high concentrations of H^+^ ions (Edge et al. 1994). However, at pH 2.5, sulfuric acid's impact was negative, with reduced germination and significantly shorter root and shoot lengths, highlighting that while sulfuric acid can be beneficial at moderate acidity, its effects are detrimental at extreme pH levels.

Hydrochloric acid, another strong inorganic acid, demonstrated a distinct pattern compared to sulfuric acid. Literature suggests that HCl can effectively break seed dormancy and promote germination of seeds with hard coats, irrespective of the acid concentration (Sharma et al. 2021; Šoch et al. 2023), which may explain the relatively higher germination rates observed across all pH levels in the HCl treatments (Figure 1). However, its impact on subsequent seedling development was clearly negative (Figure 2; Table 2). Both radish and mustard seedlings exhibited reduced biomass, smaller leaf areas, and shorter shoot and root lengths when grown under HCl, especially at the lower pH levels (Figure 2). The inhibitory effect of HCl on vegetative growth may be attributed to chloride ion (Cl^−^) toxicity or high ionic strength, which can interfere with nutrient uptake and osmotic balance (Evans et al. 2011). These results suggest that while HCl can break seed dormancy and promote germination, the environment it creates may not be conducive to seedling development, potentially impeding overall biomass accumulation and morphological development.

Acetic acid, a weak organic acid commonly found in natural acidic environments (Adeleke et al. 2017), had the most inhibitory effect on both species. At pH 2.5, germination was entirely suppressed, while at pH 4 and 5.5, germination rates under acetic acid were similar to those of the control (Figure 1), indicating that its impact is highly pH‐dependent. In terms of seedling growth, acetic acid had varied outcomes. At pH 2.5, all seedlings died by the end of the experiment for both species (Figure 2), suggesting that the high solute concentration at this pH level was lethal for developing seedlings (Taylor et al. 2021). Although acetic acid is a weak acid and only partially dissociates in water, it can still create a hyperosmotic environment that impedes water uptake, which is critical for seedling survival (Stirzaker and Passioura 1996).

At pH 5.5, mustard seedlings grown in acetic acid achieved biomass values comparable to those grown under sulfuric acid, while radish biomass remained lower but still showed some growth compared to other treatments (Figure 2A). The reduced growth, particularly for radish, may be due to acetic acid's impact on water absorption and nutrient uptake mechanisms. Despite being a weak acid, acetic acid's partial dissociation increases solute concentration, affecting cellular osmoregulation and leading to osmotic stress that likely limits overall plant development. Higher solute concentrations may negatively impact seed germination if water uptake via osmosis is reduced by decreases in water potential (Stirzaker and Passioura 1996; Taylor et al. 2021). This has been reported in high saline conditions, where seed germination is inhibited by low water potential caused by higher solute concentrations. This hinders water uptake by the germinating seeds and inhibits overall germination and subsequent seedling growth (Stirzaker and Passioura 1996; Guo et al. 2020).

For both species, leaf area and root length were consistently reduced under acetic acid treatments compared to the control, particularly at pH 4, where mustard showed a leaf area of 0.61 cm^2^ and radish reached only 1.33 cm^2^. The reduction in root growth under these conditions indicates that even though acetic acid allowed germination and initial growth, its osmotic effects and potential disruption of ion homeostasis did not support sustained seedling development. This contrasts with the effects observed under sulfuric acid, where sulfate ions might have provided a nutrient benefit that was absent in acetic acid treatments.

While our results demonstrate significant effects of acid type and pH on seedling growth, an important caveat is the potential for root exudates to buffer the pH around the roots, minimizing acidity in the immediate rhizosphere. Roots can release organic compounds that act as natural buffers (Dakora & Phillips, 2002; Nye 1981), which may have influenced the observed results, particularly in treatments where plants appeared to tolerate lower pH levels. For instance, *B. juncea* at pH 4 under acetic acid and *R. sativus* at pH 2.5 under sulfuric acid still exhibited some biomass accumulation (0.005 and 0.014 g, respectively) and morphological development such as modest root lengths (7.67 and 6.70 cm, respectively). We did not account for or measure these buffering effects, which could provide an alternative explanation for why some plants managed to grow under acidic conditions that would otherwise be expected to be detrimental.

The differential effects observed between the acids emphasize that plant responses to acid stress depend not only on pH but also on the specific chemical properties of the acids. This would suggest that wild plant communities exposed to varying types of acid deposition may exhibit diverse responses. For instance, environments subjected to industrial emissions resulting in sulfuric or hydrochloric acid deposition might see different plant community dynamics compared to those influenced by organic acids (Evans et al. 2011; Goud and Roddy 2022; Zhang et al. 2023). Our findings that hydrochloric acid, despite promoting higher germination rates, led to reduced seedling growth suggest that some plant species may experience stunted development even if they initially appear tolerant to acidic conditions. This is supported by research indicating that while acid rain at pH 2 can enhance germination, it adversely affects seedling development (Fan and Wang 2000). Similarly, inhibition of root growth could have long‐term implications for a plant's ability to absorb water and nutrients, even if initial germination is successful. Conversely, sulfuric and acetic acids, which supported better root and shoot growth, may allow certain species to maintain more balanced growth under acidic conditions, depending on the pH. A better understanding of the differential impacts of acid types on various plant growth stages and biological processes can inform more targeted management practices, aiming to maintain biodiversity and functionality in areas facing both natural and anthropogenic acidification.

## Conflicts of Interest

The authors declare no conflicts of interest.

## Data Availability

The data that support the findings of this study are available from the corresponding author upon reasonable request.
